# Medical Department of the University of Buffalo

**Published:** 1881-03

**Authors:** 


### MEDICAL DEPARTMENT OF THE UNIVERSITY OF BUFFALO.

On the morning of Feb. 22d the Seventh Annual Meeting of the Alumni Association of the University of Buffalo was held at the College. The President being absent, the assembly was called to order by Dr. R. Cotes, of Batavia, 1st Vice-President. The Executive Committee then presented their programme for the day's exercises, including those of the commencement, and the Treasurer, Dr. C. C. Wyckoff, also handed in his report.

### MEMBERS PRESENT.

The roll of classes being called, the following answered to their names:

Class of 1847—Dr. J. E. King, Buffalo; Dr. Wm. Ring, Buffalo.

Class of 1848—Dr. C. C. Wyckoff, Buffalo; Dr. H. Hoyt, East Aurora.

Class of 1852—Dr. John R. Cotes, Batavia.

Class of 1859—Dr. W. W. Potter, Batavia.

Class of 1860—Dr. C. H. Richmond, Livonia, N. Y.

Class of 1861—Dr. T. M. Johnson, Buffalo.

Class of 1863—Dr. A. J. Scott, Loudonville, O.

Class of 1865—Dr. H. Lapp, Clarence, N. Y.; Dr. A. H. Crawford, Corfu, N. Y.

Class of 1866—Dr. C. Diehl, Buffalo; Dr. W. C. Phelps, Buffalo.

Class of 1867—Dr. E. C. W. O'Brien, Buffalo.

Class of 1868—Dr. John Dambach, Buffalo.

Class of 1869—Dr. George W. Pattison, Buffalo.

Class of 1870—Dr. J. Sloan, Buffalo.

Class of 1871—Dr. A. H. Briggs, Buffalo; Dr. J. J. Walsh, Buffalo; Dr. D. W. Harrington, Buffalo; Dr. McNeil, Buffalo.

Class of 1873, Dr. Joseph Fowler, Buffalo.

Class of 1874—Dr. J. Wockener, Strykersville, N. Y.; Dr. E. N. Brush, Utica; Dr. Bernard Bartow, Buffalo.

Class of 1876—Dr. W. P. Clothier, Buffalo; Dr. M. B. Moody, Buffalo.

Class of 1877—Dr. F. G. Sherwood, Rush, N. Y.; Dr. A. M. Barker, Buffalo; Dr. William Sheehan, Rochester.

Class of 1878—Dr. A. R. Davidson, Buffalo; Dr. Eugene E. Storck, Buffalo; Dr. Frederick Sweetland, Angola, N. Y.

Class of 1879—Dr. F. Peterson, Buffalo.

Class of 1880—Dr. W. C. Barrett, Buffalo; Dr. C. M. Daniels, Buffalo; Dr. Samuel H. Warren, Buffalo; Dr. Frank O. Vaughan, Buffalo; Dr. Edward Clark, West Seneca; Dr. C. A. Driggs, Strykersville; Dr. D. E. Waldruff, Buffalo; Dr. Carl Guess, Buffalo.

On motion of Dr. Sheehan, the duty of nominating officers for the ensuing year was assigned to Doctors Lapp, Davidson, Phelps, Richmond and O'Brien.

The Association then adjourned until two o'clock.

In the afternoon, the President, D. A. G. Ellinwood, of Attica, called the Association to order at half past two o'clock.

The roll of classes was again called, and the Secretary reported the following additional members present:

Class of 1848—Dr. A. G. Ellinwood, Attica.

Class of 1851—Dr. Thomas S. Strong, Westfield, N. Y.

Class of 1855—Dr. G. M. Palmer, Lockport.

Class of 1856—Dr. H. D. Freeman, Smethport, Pa.

Class of 1859—Dr. H. Day, Arcade, N. Y.

Class of 1866—Dr. R. J. Menzie, Livonia; Dr. F. W. Abbott, Buffalo.

Class of 1870—Dr. A. G. Wallace, Rochester.

Class of 1872—Dr. Charles B. Knowlton, Buffalo; Dr. F. E. L. Brecht, Buffalo.

Class of 1873—Dr. F. A. Burghardt, Buffalo.

Class of 1874—Dr. W. D. Whitney, Lottsville, N. Y.

Class of 1875—Dr. Maudeville, Rochester.

Class of 1876—Dr. D. A. Ellsworth, East Otto, N. Y.; Dr. John G. Van Pelt, Varysburgh, N. Y.; Dr. I. A. M. Dike,

Class of 1878—Dr. M. D. Bideman, Buffalo.

Class of 1879—Dr. F. Jaekle, Dunkirk; Dr. W. H. Pitt, Buffalo.

Class of 1880—Dr. B. H. Grove, Buffalo; Dr. F. Brockway, Royalton.

OFFICERS FOR THE ENSUING YEAR.

Dr. Lapp, from the Committee appointed at the morning session, reported the following officers for the ensuing year, and the nominations were confirmed:

President—John R. Cotes, Batavia.

First Vice-President—S. D. Freeman, Smethport, Pa.

Second Vice-President—Wm. Ring, Buffalo.

Third Vice-President—Z. J. Lusk, Warsaw.

Fourth Vice-President—C. Diehl, Buffalo.

Fifth Vice-President—Wm. F. Sheehan, Rochester.

Secretary—A. M. Barker, Buffalo.

Executive Committee—W. C. Barrett, B. Bartow, E. C. W. O'Brien; James P. White, and John R. Cotes, members *ex-officio.*

Trustee—James S. Smith.

The exercises were opened by Prof. Julius F. Miner, in an address of welcome to the Alumni. The pleasure experienced by all in his appearance as an active member was warmly evinced, and as heartily reciprocated.

He expressed much gratification as well as surprise in having been selected for the part assigned him—that of receiving and welcoming the Alumni—and, believing it was an indication of sympathy for him in the long illness which had debarred him from participating in the duties of the last session, he was thus forcibly shown that the medical profession was a confederation to be strengthened and firmly established by the mutual good will of its members.

That "the child is father to the man" was illustrated to a degree by the Alumni of the Medical College, there being exhibited traces of strong peculiarities recognizable both in the College itself and in its offspring. If its complete history could be written much of deep interest would be furnished, and the Doctor regretted earlier steps had not been taken to preserve a careful record for future reference. He thought it still not too late to secure this desirable end, and offered valuable suggestions in the matter. Touching on the great changes which had taken place in the science of medicine even since his own graduation, he said that though novelty was the order of the day, it was doubtful whether as yet great superiority over the old methods had been attained, and thought the improved methods of treatment should afford more satisfactory results, but, as is very often the case, the practical advantages are by no means commensurate with the actual advancement in knowledge. He did not intend, however, to express opinions on medical or surgical subjects, but, while congratulating them on their honorable profession, to impress the truth that careful observation is essential to correct conclusions. Thanking the committee for the privilege conferred upon him, and with an earnest hope that the pleasure of these alumni anniversaries would linger in the memory of all, Dr. Miner retired amid enthusiastic applause.

Dr. Bernard Bartow was then introduced by the President. His paper was a most interesting and instructive one, on a method recently introduced, of making sole leather of practical use in surgery. In the management of such cases, where the chief objects of the treatment, are to secure rest for the maimed limbs or body, and to guard against the effects of gravitation and muscular contraction, he had found incalculable advantages in the employment of leather braces, as preventing those forces from becoming deforming agents. The use of leather for such purposes had heretofore been impracticable, on account of the difficulty of moulding it accurately to the form of the limb, or other part affected, because of its rigidity ; yet its desirable properties otherwise, made it highly important if possible to overcome this difficulty. He then exhibited a great variety of splints and supports, designed for various parts of the body, most of which had been in actual use, and thus proved by positive demonstration, that the rigidity of leather was capable of being completely overcome, giving it a first rank among materials available in every-day surgery. The practicability of his method, and the simple manner by which heavy sole leather may be made to assume almost any desired form was then fully illustrated, and the process by which the appliances are made, described. It will be unnecessary to detail here the adaptation of leather for these especial purposes, since the results of Dr. Bartow's study and experiment have already been thoroughly set forth by him in a recent number of this journal. It is sufficient to say that his valuable contribution to the advancement of mechanical surgery is attracting some attention, and also gained for him no little commendation from the gentlemen present.

The paper of Prof. Dormeus which was next in order will be given in a future number of this Journal.

Dr. Ellenwood, the retiring President, followed with an address on "The history of the organization of the Alumni, and their relation to Alma Mater and her faculty.

The meeting then adjourned to enjoy a lunch served in the museum of the College, to which both the faculty and the students did full justice. While there, Prof. Stoddard received gratifying testimony of the esteem in which he was held by the class lately under his charge in therapeutics and hygiene. The nature of the compliment is explained by the following notes:

TO PROF. STODDARD.

BUFFALO, Feb. 18th, 1881.

*Prof. E. V. Stoddard, M. D., University of Buffalo:*

We, the members of the class of 1881, having listened with great pleasure and profit to your lectures of the present session, and being desirous of obtaining them in permanent form for future reference, unite in requesting you to prepare and publish the same at your earliest convenience.

Most respectfully yours,

EUGENE E. BARNUM,<br>

EUGENE G. HOITT,<br>

GEORGE WALDRON. } Committee.

At the table, Dr. Chas. Cary, Secretary of the Faculty, read the following

REPLY.

UNIVERSITY OF BUFFALO,<br>

Feb 21st, 1881.

*Messrs. Barnum, Hoitt, and Waldron, Committee:*

GENTLEMEN—Your communication requesting the publication of the course of lectures delivered during the session just completed in the Department of Therapeutics and Hygiene is received. In reply I can only say that I will endeavor to comply with your request before the commencement of the next ensuing course. Thanking you for your kindly expressions of appreciation, I remain

Very sincerely yours,

E. V. STODDARD.

In the evening the commencement exercises were held at St. James Hall, attracting a large audience of ladies and gentlemen, and passing off in a very satisfactory manner to all engaged.

After an overture by Wahle's orchestra, a brief prayer was offered by Bishop Coxe, at the conclusion of which Dr. White, the President of the Faculty, administered the usual oath to the graduates. They were called to the stage in classes of ten, and received by Vice-Chancellor O. H. Marshall, who conferred on them the degree of doctor of medicine.

The following is a list of their names, residences, and the subject of their theses.

Henry Adward Latz, Buffalo, N. Y., Typhoid Fever.

Henry Hinman Coleman, Kings Ferry, N. Y., Rubeola.

Thomas Henry Carroll, Smethport, Pa , Synovitis.

Walter Hamilton Lockerby, East Newfield, N. Y., Superstition vs. Medicine.

Eugene Everett Barnum, Murray, N. Y , Carcinoma of the Stomach.

Charles Henry Woodard, Blood's Station, N. Y., Physiological action of Alcohol.

William Bell Brown, Bath. N. Y., A case of Periosteal Sarcoma.

George Waldron, Rush, N. Y., Water as an internal and prophylactic agent.

William Byron Johnston, Ellicottville, N. Y , Laryngitis with Exudation.

William Henry Vincent, Salamanca, N. Y., Erysipelas.

N. Sanford Messenger, Breeseport, N. Y., Scarlatina.

Charles Mark Brasted, Hornellsville, N. Y., Tuberculosis Meningitis.

Israel Bidaman, Buffalo, N Y., The scope and application of Philosophical Medicine.

Eugene Gorham Hoitt, Port Jervis, N. Y., Entero-mesenteric fever.

William Abbott Hubbard, Meridan, N. Y., Progressive muscular atrophy.

Jesse Orimal Randall, Machias, N. Y., Acute Peritonitis.

William Wilbur Williams, Waterport, N. Y., Scarlatina.

Herbert Browning Love, Jamestown, N. Y., Hypodermic Indication.

Joseph Skerm Laning, Woodsville, N. Y., Treatment of Abortion.

William Henry Parker, Clarence, N. Y., Scarlatina.

Alfred Rufus Crain, Richfield Springs, N. Y., The administration of Anæsthetics.

George Dawson York, Lyons, N. Y., Pneumonitis

Mary Elizabeth Runner, Buffalo, N. Y., Heredity.

Laurentine Raochel, Rochester, N. Y., Infantile Paralysis.

Homer Truman Jackson, Higginsville, N. Y., Progressive Medicine.

Robert Newland Blanchard, Jamestown, N. Y., Physical and mental hygiene.

Carlton Cassius Frederick, Buffalo, N Y., Nerves and nerve terminations.

Charles Grover Plumb, Lyons, N. Y., Typhoid Fever.

William Alfred Hobday, Buffalo, N. Y., Astigmatism.

Luther William Tarbox, South Dayton, N Y., Ovulation and Menstruation.

William Martin, Fort Erie, Canada, Typhoid Fever.

Charles Clarence Winsor, Jamestown, N. Y., Encephaloid Tumors.

James Middleditch, Gilbertsville, N. Y., Post Partum Hemorrhage.

John Joseph Birmingham, Batavia, N Y., A superficial examination of the urine at the bedside.

Adelbert Gideon Bush, Erin, N. Y., Fever.

Lyman Rexford Raymond, Evans, N. Y., Malarial Fever.

John B. Hoyer, Middleport, N. Y., Abortion.

Peter Stockschlæder, Rochester, N. Y., Scarlatina.

Charles Brooks Scott, Landonsville, O., Diseases and Injuries to Joints.
Nicholas Frederick Kiefer, Buffalo, N. Y., Facts concerning the blood.
George Ferguson Hunter, Holly, Mich., Carcinoma or Cancer.
Harvey Leverne James, Medina, N. Y., Poisons.
Clarence Le Roy Jones, Evans' Mills, N. Y., Uterine Hydatids.
John Wesley Bickford, Somerset, N. Y., Anæmia.
William Henry De Puy, Rochester, N. Y., Post Partum Hemorrhage.
Irving Miller Snow, Buffalo, N. Y., Inflammation.
Myron Caspar Hawley, East Randolph N. Y., Diphtheria.
John Westley Pease, Lockport, N. Y., Diphtheria.

The business portion of the programme being then finished, Prof. E. M. Moore of Rochester was introduced, and delivered the address to the graduating class. He first called attention to the astonishing progress medical science had made since the beginning of the world's history, and presented some interesting thoughts on the civilization of the ancient Egyptians, their proficiency in astronomy, arts, &c., being concisely commented upon. He then referred to the universal custom among them of embalming their dead, and drew the conclusion that this practice had been a formidable bar to the advancement of medical knowledge, since it actually arrested on the very threshold, most essential investigations. The father of medicine was Hippocrates, the Greek, whose teachings may be considered to have established the scientific medical school. After him came Galen, who produced over three hundred treatises on medical subjects, but though the ideas and methods of these two eminent physicians, soon filled the civilized world with disciples, the progress of medical science was not rapid. From the sixteenth century, however, the development of intellectual power in general did not allow the knowledge of medicine to lie dormant. Great scholars and thinkers enriched the field of anatomical and surgical study, among the earliest of whom was Andreas Vesalius, who dissected not only the bodies of men, but of animals, thus laying the foundation of comparative anatomy. After Vesalius came Ambrose Pare, who changed the practice of cauterization in arresting hemorrhage, for the employment of the ligature. Nor must Fallopius nor Harvey be forgotten when enumerating the names of those to whom medicine owes its marvelous march of improvement in recent years. The facts enumerated tended to show that it was only after science had broken the bands of superstition, and investigators claimed for themselves the most unlimited freedom of opinion, that medical science made any advancement.

Bishop Coxe next adressed the Alumni with his customary fluency. He remarked that all present, watched with interest the progress of the Medical College, an institution in which just pride was felt. It had already attained a fair degree of success, and its field of usefulness was undoubtedly destined to become still more honorable and extended. As the manufacturing interests of Buffalo increased, there would be an increasing demand by its growing population for medical and surgical service. He spoke of the general healthful condition of Buffalo, but admitted it had some stains on its reputation, and hoped physicians would unite in abating several of its most obnoxious nuisances. Subsequently he briefly touched upon a few of the problems agitating the popular mind at present, questions which the new-born generation of physicians would doubtless be called upon to consider and decide. Recommending them to cultivate a taste for writing and to put their best thoughts into literature, which might be of service to the public at large, the Bishop concluded by a beautiful allusion to the "Good Physician," whose tender and beneficial presence upon earth is so admirably described in St. Luke.

The evening proceedings closed with this address, and were followed by a supper at the Tifft House, where pleasant social intercourse was enjoyed by all after the more serious duties of the day. At the proper time these toasts were proposed and responded to:

Our Alma Mater, Dr. James P. White; The Alumni Association, Dr. T. R. Cotes; The Medical Profession, Dr. W. W. Potter; The Medical Press, Dr. Lucien Howe; The Secular

Press, Mr. James N. Mathews; Psychological Medicine, Dr. Judson P. Andrews; The Orators of the Evening, Prof. E. M. Moore; Medical Education, Dr. Thomas F. Rochester; The Graduating Class, Dr. Thomas H. Carroll.

Other toasts were proposed informally, and responses made by the following gentlemen: The Buffalo Medical Club, Dr. J. W. Keene; Public Health, Dr. H. Briggs; Foreign Universities, Dr. Herman Mynter; The Curators, Dr. Richmond.

Altogether the graduating exercises were eminently satisfactory, and the meeting of the Alumni gratifying in a social and professional point of view.

